# Objective and Subjective Factors as Predictors of Post-Traumatic Stress Symptoms in Parents of Children with Cancer – A Longitudinal Study

**DOI:** 10.1371/journal.pone.0036218

**Published:** 2012-05-02

**Authors:** Annika Lindahl Norberg, Ulrika Pöder, Gustaf Ljungman, Louise von Essen

**Affiliations:** 1 Psychosocial Oncology and Supportive Care, Department of Public Health and Caring Sciences, Uppsala University, Uppsala, Sweden; 2 Childhood Cancer Research Unit, Department of Woman and Child Health, Karolinska Institutet, Stockholm, Sweden; 3 Caring Sciences, Department of Public Health and Caring Sciences, Uppsala University, Uppsala, Sweden; 4 Department of Women's and Children's Health, Pediatric Oncology, Uppsala University, Uppsala, Sweden; Federal University of Rio de Janeiro, Brazil

## Abstract

**Background:**

Parents of children with cancer report post-traumatic stress symptoms (PTSS) years after the child's successful treatment is completed. The aim of the present study was to analyze a number of objective and subjective childhood cancer-related factors as predictors of parental PTSS.

**Methods:**

Data were collected from 224 parents during and after their child's cancer treatment. Data sources include self-report questionnaires and medical records.

**Results:**

In a multivariate hierarchical model death of the child, parent's perception of child psychological distress and total symptom burden predicted higher levels of PTSS. In addition, immigrants and unemployed parents reported higher levels of PTSS. The following factors did not predict PTSS: parent gender, family income, previous trauma, child's prognosis, treatment intensity, non-fatal relapse, and parent's satisfaction with the child's care.

**Conclusions:**

Although medical complications can be temporarily stressful, a parent's perception of the child's distress is a more powerful predictor of parental PTSS. The vulnerability of unemployed parents and immigrants should be acknowledged. In addition, findings highlight that the death of a child is as traumatic as could be expected.

## Introduction

Not surprisingly, most parents of children in treatment for a malignant disease perceive the situation as severely stressful. Indeed, parental reactions indicate that the situation for many involves an existential crisis, i.e. a psychological trauma inducing reactions of traumatic stress. Reactions of traumatic stress are exhibited not only immediately following the disclosure of the diagnosis [1], [2], but also years after completion of the treatment [3], [4]. In unfavourable cases the reactions can develop into a persistent, distressing syndrome: post-traumatic stress disorder (PTSD).

Briefly, post-traumatic stress is one of the possible adverse effects of a psychological trauma. Psychological traumas are events that involve actual or threatened death or serious injury, or a threat to the physical integrity of oneself or others [5]. Certain situational factors, e.g. socioeconomic status and minority status [6] are associated with increased vulnerability to post-traumatic stress symptoms (PTSS) and PTSD.

To prevent the development of PTSS and PTSD, interventions may choose either of two foci: early adequate support to vulnerable individuals, or eliminating the occurrence of traumatic events. For most traumas, the latter is not possible. However, in the case of serious illness, it may be possible to modify aspects of the medical care and thus mitigate the traumatic impact, beside the psychological support to individuals.

Several studies during the past decades have explored predictors of PTSD and PTSS in parents of children with cancer, but the findings seem somewhat inconsistent. For example, being the parent of a child on treatment for a more severe illness [7] or a relapse [8] seems to produce severe stress. At the same time, neither the experience of a more severe illness [9] nor the experience of a relapse [3], [4] appear to be associated with PTSS after completion of successful treatment. Kazak, Boeving, Alderfer, Hwang, and Reilly [2] demonstrated a relationship between treatment intensity and concurrent arental traumatic stress, while Kazak, Stuber, Barakat, Meeske, Guthrie, and Meadows [10] found no association between treatment intensity and parents' PTSS some years after the end of successful treatment. In sum, previous findings indicate that certain disease-related factors cause immediate traumatic stress reactions, however, do not increase the long-term risk for PTSS or PTSD among parents of childhood cancer survivors.

Leaning on appraisal theory [11], one could expect subjectively appraised disease-related factors to be stronger predictors of PTSS than objective factors. This hypothesis has been partly confirmed, demonstrating associations between parents' perceptions of threat to the child's life and PTSS [10], [12], [13]. However, subjective factors and PTSS are typically assessed simultaneously, which prevent analyses of causality.

In conclusion, for parents several events during a child's cancer illness and treatment may be severely stressful and bring about immense distress, but they may still not be traumatic enough to cause post-traumatic stress, i.e. reactions lasting long after the event occurred. This combination of minor hassles, severe but manageable stressors and traumatic experiences puts a challenge to the research on traumatic stressors and predictors of post-traumatic stress symptoms in the cancer setting.

A drawback of previous studies may be that investigated potential predictors of PTSS might have been too general (e.g. “length of treatment”). Moreover, the study groups often comprise cross-sectional samples including parents at the time of their child's diagnosis and up to about 10 years after the end of treatment. This reduces the possibility to analyze subtle features of the situation, since most disease-related stressors can be assumed to be present for some parents, in the past for some, and still in the future for others. In addition, cross-sectional designs have often been used, preventing prediction.

In order to develop and deliver adequate preventative interventions targeting PTSS in parents of children with cancer we need to advance the knowledge about which critical event, or events, that are involved in the occurrence, development and/or maintenance of PTSS. In an attempt to add to the existing knowledge on this matter the present study uses an empirical, theoretical, and clinical basis to identify possible predictors of PTSS in parents of children with cancer. Explicitly, we addressed stressors pointed out as important in previous studies of PTSS among parents of childhood cancer patients and survivors, and we have strived to interpret these stressors from a theoretical basis as well as using the clinical experience of paediatric oncologists, psychologists and nurses. The aim was to analyze the predictive power of a number of objective and subjective cancer-related factors for PTSS among parents of children with cancer, one year after the end of successful treatment. General risk and resilience factors that may be relevant to this situation were also considered. Specifically, the analyses concerned objective cancer-related factors, subjective cancer-related factors, and demographic and socioeconomic factors.

## Methods

The results are based on data collected within an ongoing Swedish project investigating occurrence and development of post-traumatic stress disorder among parents of children with cancer. The project has a longitudinal design with seven assessments (T1–T7): one week (T1), two months (T2), and four months (T3) after diagnosis and one week (T4; parents of deceased children were not included at this point), three months (T5), and one year (T6) after end of successful treatment or death of the child. A prolonged schedule was applied when a child had a stem cell transplant: six months (T4), nine months (T5), and one and a half year (T6) after transplant. A T7-assessment 5 years after successful treatment or death is ongoing, and is not included in this study.

### Ethics statement

Ethical approval was obtained from the local ethics committees at the respective faculties of medicine in Gothenburg, Linköping, Umeå, and Uppsala, and included approval of the use of oral consent. At the time of the study it was standard to use oral consent (as opposed to written consent) in telephone survey studies. The investigation was conducted according to the principles expressed in the Declaration of Helsinki.

### Sample

Parents of children treated for cancer at four of the six Swedish paediatric oncology centres, Gothenburg, Linköping, Umeå, and Uppsala, were consecutively included during 2002–2004, eighteen months at each centre. Inclusion criteria were Swedish and/or English speaking parents (including step parents) of children 0–18 years, diagnosed with cancer for the first time and scheduled for chemo- and/or radiotherapy.

Three hundred and twenty five parents were invited to participate, of whom 66 refused participation yielding a response rate of 80%. At the subsequent assessments parents were approached if the child was on curative treatment (T2, T3), had ended a favourable treatment (T4), and had ended a favourable treatment or had died (T5, T6). The present study includes all parents who participated at T6 (N = 224; response rate 69%). Among those, all participated at T1, 220 at T2 (3 were temporarily excluded, 1 refused participation), 194 at T3 (28 were temporarily excluded, 2 temporarily refused participation), 200 at T4 (21 were temporarily excluded, 3 temporarily refused participation), and 217 at T5 (7 temporarily refused participation). One hundred seventy four parents participated at all assessments, T1–T6. For a presentation of parent and child characteristics, see Table 1. More fathers than mothers worked full time before the child's diagnosis (69% vs. 31%). The families lived on an average of 145 kilometres from the respective centre (SD 113, range 1–600 kilometres).

**Table 1:** Parent (N = 224) and child characteristics.

*T1* a	%	n
Age of parent, years		
<30	9	(21)
30–39	53	(118)
≥40	38	(85)
Education		
≤Nine year elementary	12	(27)
Upper secondary	54	(120)
University	34	(77)
Parent of daughter/son	47/53	(106/118)
Age of child, year		
0–3	23	(52)
4–7	31	(69)
8–12	26	(59)
13–18	20	(44)
Sibling/s, yes	92	(207)
Diagnosis		
Leukaemia	40	(89)
Lymphoma	18	(41)
CNS tumour	12	(27)
Other solid tumour	30	(67)
***T1 to T6*** b		
Transplant	16	(36)

a T1: one week after the child's diagnosis.

b T6: one year after end of treatment or death/1.5 year after transplant.

### Data collection

Parents answered questions through structured telephone interviews about PTSS (T1–T6), and the independent variables: perceptions of the child's symptoms (T1–T6), satisfaction with the child's care (T1–T4, T5–T6 if applicable), and demographics (T1–T6; e.g. occupational status, annual household income, ethnicity, and previous trauma experience). A nurse at the respective centres collected medical data for the children from the medical charts (T1–T6).

#### Post-traumatic stress symptoms

Data on PTSS were collected with the PTSD Checklist Civilian Version (PCL-C) [14], translated by our research group into Swedish using a forward-backward procedure [15]. The PCL-C consists of 17 items organized in three subscales. Eight items (item 1–8) are keyed to a specific trauma, in this study to the child's cancer disease. The respondent is asked to report how much he or she has been bothered by each item during the last month (at T1 during the last week) on a 5-point scale ranging from not at all (1) to extremely (5). The subscales correspond to the three symptom clusters of PTSD according to the DSM-IV [16]: re-experience (5 items; Cronbach's alpha (α) in this sample at T6: .88), avoidance (7; α .82), and hyper-arousal (5; α .89). The total score ranges from 17 to 85 (α .94). The total scale score at T6 was used as the outcome measure of PTSS in the present study.

#### Perceptions of the child's symptoms

Parents answered a modified version of the Memorial Symptom Assessment Scale for children (MSAS 10–18) [17], [18], translated into Swedish by our research group using a forward-backward procedure [15]. The questionnaire is organized in three subscales: the psychological symptoms subscale (PSYCH), the physical symptoms subscale (PHYS) and the Global Distress Index. In addition a score of total number of symptoms is calculated. For this project, the questions were modified to be answered by parents according to their perceptions of their child's symptoms. In accordance with previous findings [18], the instrument used in this study includes questions about headache and hair-loss in addition to the original 30 items; however, these are not included in the PSYCH subscale or the PHYS subscale. For each symptom parents were asked to assess whether it had been present during the last week, and if so, to rate it according to frequency, intensity, and distress. Answers were provided on Likert scales: frequency, from almost never (1) to almost always (4); intensity from slight (1) to very severe (4), and distress from not at all (0) to very much (4). Symptoms reported as not present are given a value of 0 for frequency, intensity, and distress. The T3-assessments of the PSYCH subscale (α .77), the PHYS subscale (α .81), and total number of symptoms are used as potential predictor variables of PTSS in the present study. This assessment point was selected since symptoms experienced at this time can be assumed to be more stressful for parents compared to when experienced earlier during the treatment when symptoms are more common and therefore more expected. A higher score reflects more symptoms.

#### Satisfaction with the child's care

Parents' satisfaction with the child's care was measured with the CASC SF Version 4.0 [19], consisting of 32 questions, with acceptable test-retest reliability. The original version was constructed as a self-report instrument, however, in this study answered by parents according to their opinion of the child's care. At T1 parents answered the questions since the child's diagnosis whereas at T2 to T6 since the last interview. Responses were provided on 5-point scales ranging from very poor (1) to excellent (5). The questionnaire is organized in eleven multi-item scales and three single items [19]. In the present study, the single item general satisfaction was used as a potential predictor variable of PTSS.

#### Medical data

Medical data were collected from the child's medical records. An experienced paediatric oncologist (the 3^rd^ author; blind for parental PTSS scores) estimated child's prognosis and treatment intensity.

#### Prognosis

Diagnosis, localization, stage of the disease and risk group at initial diagnosis, was used to estimate prognosis, based on data for the Nordic countries concerning the probability of 5-year survival considering given conditions [20]. Since 75% was the average childhood cancer survival in Sweden at the time when prognosis was estimated, this value was used as the cut-off to form two categories: ≥75% vs. <75% chance of 5-year survival.

#### Treatment intensity

Treatment intensity was estimated as high intensity vs. not high intensity. High intensity included the following diagnoses and protocols: AML (all protocols), ALL (extra intensive/very intensive protocols and Philadelphia positive), Ewing sarcoma (all protocols), Osteosarcoma (all protocols), B-cell lymphoma, Neuroblastoma (high risk), HIT (Hirntumor)-protocol, SIOP 4 PNET-protocol, BMT/SCT, and other treatments for high risk groups. All other treatments were assigned to the category not high intensity.

#### Treatment complications

Data from the child's treatment in total, T1–T4, were collected regarding: number of hospitalizations due to infections; number of days (>2 continuous days) in intensive care; number of blood transfusions; number of antibiotic treatments; relapse, and whether the child died from his/her disease.

#### Demographic and socioeconomic factors

Data on employment status (working full time or part time/being a student, vs. being unemployed/on long-term sick-leave); ethnicity (persons born outside the Nordic countries (immigrants) vs. persons born in the Nordic countries); annual household income (up to 33 500 Euro vs. more than 33 500 Euro), and whether the respondent had experienced any traumatic event prior to the child's cancer diagnosis, which he/she believed could influence present reactions (the specific characteristics of the trauma were not analysed).

### Procedure

Parents who met the inclusion criteria received written and oral information about the study from a coordinating nurse at the respective centre within two weeks after the child's diagnosis (M = 4 days after diagnosis). Thereafter oral informed consent was asked for over telephone by one of two interviewers (M = 7 days after diagnosis). Permission to contact the parent at the next data collection was acquired at the end of each interview. The interviews were conducted over the telephone on an average of 8 (T1), 61 (T2), and 120 days (T3) after the child's diagnosis, and on an average of 13 (T4; no bereaved parents), 96 (T5) and 374 days (T6) after the end of successful treatment or death of the child. In the case of transplant, the assessment was postponed six months at T4, T5 and T6.

### Data management and analyses

All independent variables that univariately demonstrated associations with PTSS (univariate linear regression) were included in a Hierarchical multivariate model. In this model families (children) were considered clusters of parents. A hierarchical model was considered the most feasible for the kinds of variables studied. Inclusion as well as measurement of some variables was based on the child treated for cancer. The child could have more than one parent who might or might not yield independent measurements of one another. Therefore, a non-hierarchical model could only have been built either by combining the parental measures for each child, giving the number of observations equal to the number of children in the study, or by counting child measurements several times which leads to a possibility of spurious significances. Moreover, the clustering was found significant in model comparisons and as such was needed in order to give correct results on both parent-level and child-level measurements.

## Results

Descriptive statistics for the independent and dependent variables are presented in Table 2.

**Table 2:** Descriptive statistics for the independent and dependent variables (n = 224).

	% (n)	mean (SD); range
**Sociodemographic and parent variables**		
Parent gender: mother or stepmother	50% (112)	n.a.
Parent ethnicity: born in the Nordic countries	96% (214)	n.a.
Parent employment status at diagnosis: employed/student	87% (194)	n.a.
Family income at diagnosis: more than 33 500 Euro annually^a^	82% (184)	n.a.
Parent traumatic experience prior to diagnosis	31% (70)	n.a.
**Parent's perception of child's treatment/cancer**		
Parent satisfaction with care at T1	n.a.	89 (15); 25–100
Parent perception of child psychological symptoms at T3 (MSAS PSYCH)^b^	n.a.	.61 (.65); 0–3.33
Parent perception of child physical symptoms at T3 (MSAS PHYS)^b^	n.a.	.71 (.62); 0–3.15
Parent perception of child's number of symptoms at T3^b^	n.a.	9 (5); 0–26
**Child medical variables** c		
Child prognosis: ≥75%	53% (118)	n.a.
Child treatment intensity: high	51% (115)	n.a.
Child with non-fatal relapse	8% (18)	n.a.
Death of the child	17% (37)	n.a.
Antibiotic treatments of the child^d^	n.a.	4 (4); 0–22
Hospitalizations of the child due to infections^d^	n.a.	2 (2); 0–8
Blood transfusions of the child^d^	n.a.	11 (17); 0–122
Child treated at ICU, number of days^d^	n.a.	1 (2); 0–11
**PTSS**		
PCL-C score	n.a.	28.6 (12.0); 17–75

PTSS: Post-traumatic stress symptoms; PCL-C: PTSD Checklist Civilian Version; n.a.: not applicable.

a Missing data: five parents.

b Missing data: 30 parents (mainly because their children were off treatment at T3).

c For child variables, the values indicate percentage/number of parents.

d Total during the child's cancer treatment.

### Univariate associations

Higher levels of PTSS 12 months after completed successful treatment or death, or 18 months after transplant were reported by mothers, immigrants, parents who had experienced a previous trauma, unemployed, and parents with a lower income (Table 3). Furthermore, higher levels of PTSS were associated with a child's poorer prognosis, more intense treatment and the death of a child, and by a parent's perception of the child's status during treatment regarding psychological symptoms, physical symptoms, and total number of symptoms. The factors explaining the highest amount of variance in parental PTSS were child's psychological symptoms (18.6%), child's total number of symptoms (17.4%), parent gender (16.7%), and death of the child (12.6%).

**Table 3:** Univariate associations between potential predictors and parental PTSS 12 months after completed treatment or death, or 18 months after transplant (Univariate linear regression; n = 224).

	R^2^
**Sociodemographic and parent variables**	
Parent gender	.167*
Parent ethnicity	.044**
Parent employment status at diagnosis	.073***
Family income at diagnosis^a^	.020*
Parent traumatic experience prior to diagnosis	.029*
**Parent's perception of child's treatment/cancer**	
Parent satisfaction with care at T1	.019 ^n.s.^
Parent perception of MSAS PSYCH at T3^b^	.186***
Parent perception of MSAS PHYS at T3^b^	.082***
Parent perception of MSAS number of symptoms at T3^b^	.174***
**Child medical variables**	
Child prognosis	.025*
Child treatment intensity	.031**
Child with non-fatal relapse	.0001 ^n.s.^
Death of the child	.126***
Antibiotic treatments of the child	.001 ^n.s.^
Hospitalizations of the child due to infections	.002 ^n.s.^
Blood transfusions of the child	.007 ^n.s.^
Child treated at ICU, number of days	.015^-n.s.^

PTSS: Post-traumatic stress symptoms.

* p<.05;

** p<.01;

*** p<.001;

n.s. not significant.

a Missing data: five parents.

b Missing data: 30 parents.

### Hierarchical multivariate model

All variables that demonstrated univariate associations with PTSS were included in the multivariate hierarchical model. In addition, satisfaction with care during the first weeks of treatment, and non-fatal relapse were considered of particular interest, and were included in the model despite lack of significant univariate associations with PTSS.

Parents who reported more psychological symptoms or a higher number of symptoms for their child four months after the diagnosis as well as parents who had lost their child were more likely to report PTSS 12 months after completed successful treatment or death, or 18 months after transplant (Table 4). Moreover, child physical symptoms demonstrated a negative association, indicating higher levels of PTSS in parents who reported less physical symptoms for their child four months after the diagnosis. In addition, being an immigrant or unemployed at the time of the child's diagnosis predicted PTSS.

**Table 4:** Associations between potential predictors and parental PTSS 12 months after completed treatment or death, or 18 months after transplant (Hierarchical multivariate model; n = 224).

	Unstandardized B
Parent gender	−2.162 ^n.s.^
Parent ethnicity	12.010**
Parent trauma prior to diagnosis	0.836 ^n.s.^
Parent employment status at diagnosis	−5.389*
Family income at diagnosis^a^	1.072 ^n.s.^
Child prognosis	−0.623 ^n.s.^
Child treatment intensity	0.877 ^n.s.^
Parent satisfaction with care at T1	−0.087 ^n.s.^
Parent perception of MSAS PSYCH at T3^b^	3.688*
Parent perception of MSAS PHYS at T3^b^	−5.007*
Parent perception of MSAS number of symptoms at T3^b^	0.765*
Child with non-fatal relapse	4.622 ^n.s.^
Death of the child	9.690***

PTSS: Post-traumatic stress symptoms.

* p<.05;

** p<.01;

*** p< .001;

n.s. not significant.

a Missing data: five parents.

b Missing data: 30 parents.

## Discussion

Post-traumatic reactions have been reported by parents of children with cancer in numerous studies during the past decades. However, research has not revealed which aspects of the cancer experience that are challenging enough to cause these reactions. Previous studies have rarely identified any associations between disease-related factors and long-term PTSS among parents. Reasons could be that the cancer diagnosis per se is the traumatic event, and that the specific experiences attached to the individual case are of less importance, or that previous studies did not cover the key potentially traumatic events. In the present study we aimed at extending the knowledge about which factors that predict long-term PTSS among parents of children struck by cancer.

A medically more troublesome disease trajectory is related to parental stress [2], [3]. However, in the multivariate model a finally fatal disease was the only objective disease-related factor that demonstrated predictive power for PTSS.

Not surprisingly, the death of a child was one of the two strongest predictors of parental PTSS, also when analyzed together with other potentially traumatic aspects of childhood cancer. The death of a loved one is a truly traumatic event. However, a resemblance between PTSS and grief may contribute to an overestimation of PTSS reported by bereaved persons [21]. Nonetheless, we conclude that parents who meet this tragic ending are vulnerable to post-traumatic stress and/or prolonged grief [22], [23].

For parents whose child had survived their disease at the time when PTSS were assessed, none of the objective medical events targeted in the present study seemed to be traumatic enough to produce lingering post-traumatic stress symptoms. Since fear of relapse is known to be a prominent stressor [24], one could assume that an actual relapse should be such an event. Yet, non-fatal relapse did not predict PTSS. This is in line with results from a previous Swedish cross-sectional study [25], although findings by others have been ambiguous about this. Jurbergs et al. [8] have reported that a child's cancer-related relapse predict parental traumatic stress symptoms. However, their cross-sectional sample most likely includes parents in acute crisis, and is not comparable with the present sample. Medical complications associated with the child's treatment, such as serious infections, ICU treatment et cetera are definitely stressful when occurring [26], but do not seem to produce enduring PTSS.

Poor prognosis and intense treatment evidently correspond with a fatal disease development. Accordingly, the death of the child may be the underlying factor explaining later post-traumatic stress symptoms in those parents, while general factors accompanying a more problematic disease and treatment do not automatically produce lingering PTSS.

Every parent can certify that experiencing that one's child suffers arouses parental distress [27] and the findings indicate that parents' perceptions of their children's suffering predict long-term PTSS. In the multivariate model, the perceived number of child symptoms and child psychological distress demonstrated a significant predictive power for PTSS. However parental perceptions of physical symptoms in the child seemed to be protective, when its impact on PTSS was analyzed together with other predictors. This result is difficult to interpret, and therefore we refrain from speculations. The implication of this needs to be further explored.

It is well documented that unemployment is a risk factor for PTSS as well as for other mental health problems [23], [28]. In our categorization, non-employed includes being job-seeking and unemployed and/or on long-term sick-leave at the time of the child's diagnosis. Tentatively we suggest that the normalizing milieu including support from the social network of a workplace may buffer against mental health problems.

Supporting previous findings [25], being an immigrant was shown to substantially predict PTSS. A reasonable assumption could be that immigrants more often than non-immigrants are troubled by consequences of previous trauma, making them more vulnerable to an additional trauma. However, when analyzed in the multivariate model, previous trauma did not predict PTSS. Instead, the explanation may be sought in insufficient social support, and cultural differences in the connotations of illness and the communication with the health care staff [29], [30]. Noteworthy is that gender did not predict PTSS when analyzed with other variables, indicating that mothers and fathers alike may develop long-term post-traumatic stress in the face of childhood cancer.

Certain limitations are attached to the present study. Firstly, we addressed only some of several possible predictors of PTSS in parents of children with cancer. Through the selection or the means of assessing those factors we may have failed to spot important issues, regarding for example the individual child's reactions to specific treatment procedures. In addition, any previous traumata and their consequences should be analyzed in more detail in future studies. Moreover, assessing PTSS through self-report questionnaires is a cost-effective approach in large samples like this, but a face-to-face clinical interview would most certainly capture the concept in a more correct way. Strengths of the study include its population based longitudinal design and relatively large sample.

It may seem inconsistent to include factors in the multivariate model, which had not shown association with PTSS in the univariate analyses. However, the factors satisfaction with care and non-fatal relapse were included for an explorative purpose for the following reasons: Satisfaction with care was considered to potentially indicate a feeling of security and a safe environment, which could be a protective factor against post-traumatic stress, and non-fatal relapse was considered to potentially indicate a re-traumatization, which could be a risk factor for post-traumatic stress (repeated trauma has been shown to be a risk factor for PTS). Although these factors were not related to PTSS in the univariate analyses, there was a hypothetical possibility for impact in the hierarchical clustered model.

In conclusion, parental traumatic stressors in childhood cancer seem not to be found in treatment complications, but in parents' subjective perceptions of their child's suffering. Relapse may be a severe stressor, but for those whose child survives, the fear evoked by a relapse typically subsides and does not leave post-traumatic stress symptoms. Moreover, although the chronic stress of present problems and feared future difficulties bring about exhaustion in parents [31], the death of a child remains the ultimate trauma in the childhood cancer experience. In addition, certain demographic factors previously recognized as risk factors for mental health problems point to a vulnerability to PTSS in parents of children with cancer: being an immigrant and being unemployed. We may well assume that these more vulnerable parents are less apt to ask for support from the paediatric medical service.

There is reason to emphasize the clinical implications of the present findings. Parents' perceptions of their child's situation should always be considered since these may have a significant impact on long-term parental mental health. Parents' mental health may in turn have an impact on their child's mental health as well as on the communication with the health care professionals. We cannot presuppose that parents of children with a medically unproblematic journey through disease and treatment do not run a risk of lasting post-traumatic stress. In addition, we should be as attentive to fathers' long-term distress as we are to mothers'. Moreover, when a parent loses his or her child, we should keep in mind that signs of PTSS or prolonged grief may indicate a condition that requires professional psychological treatment.
